# Digital PCR Panel for Sensitive Hematopoietic Chimerism Quantification after Allogeneic Stem Cell Transplantation

**DOI:** 10.3390/ijms17091515

**Published:** 2016-09-09

**Authors:** Tanja Stahl, Caroline Rothe, Manja U. Böhme, Aloisa Kohl, Nicolaus Kröger, Boris Fehse

**Affiliations:** 1 Department of Stem Cell Transplantation, University Medical Center Hamburg-Eppendorf, 20246 Hamburg, Germany; t.stahl@uke.de (T.S.); aloisakohl@hotmail.com (A.K.); n.kroeger@uke.de (N.K.); 2 Biotype Diagnostic GmbH, 01109 Dresden, Germany; c.rothe@biotype.de (C.R.); m.boehme@biotype.de (M.U.B.)

**Keywords:** chimerism, minimal residual disease, digital PCR, allogeneic stem cell transplantation

## Abstract

Accurate and sensitive determination of hematopoietic chimerism is a crucial diagnostic measure after allogeneic stem cell transplantation to monitor engraftment and potentially residual disease. Short tandem repeat (STR) amplification, the current “gold standard” for chimerism assessment facilitates reliable accuracy, but is hampered by its limited sensitivity (≥1%). Digital PCR (dPCR) has been shown to combine exact quantification and high reproducibility over a very wide measurement range with excellent sensitivity (routinely ≤0.1%) and thus represents a promising alternative to STR analysis. We here aimed at developing a whole panel of digital-PCR based assays for routine diagnostic. To this end, we tested suitability of 52 deletion/insertion polymorphisms (DIPs) for duplex analysis in combination with either a reference gene or a Y-chromosome specific PCR. Twenty-nine DIPs with high power of discrimination and good performance were identified, optimized and technically validated. We tested the newly established assays on retrospective patient samples that were in parallel also measured by STR amplification and found excellent correlation. Finally, a screening plate for initial genotyping with DIP-specific duplex dPCR assays was designed for convenient assay selection. In conclusion, we have established a comprehensive dPCR system for precise and high-sensitivity measurement of hematopoietic chimerism, which should be highly useful for clinical routine diagnostics.

## 1. Introduction

Allogeneic hematopoietic stem cells transplantation (SCT) represents an established curative treatment for various malignant and non-malignant hematological disorders such as leukemia, aplastic anemia, multiple myeloma and lymphoma [1]. Replacement of recipient by donor hematopoiesis is key for the success of SCT. Therefore, the ratio of donor to recipient blood cells, the hematopoietic chimerism, needs to be closely observed, particularly in the early engraftment phase. Moreover, chimerism monitoring provides the opportunity for therapeutic intervention even in the absence of cytogenetic, morphologic or clinical evidence of disease recurrence [2,3].

Different techniques are available for chimerism analysis, but PCR-based analysis of short tandem repeats (STRs) is most widely used [4] due to its very good accuracy and reproducibility. Unfortunately, STR-PCR (“STR”) has a comparatively low detection threshold (approximately 1%–5%) limiting its usefulness in the early detection of impending cytological relapse. In contrast, real-time quantitative PCR (qPCR), using deletion/insertion polymorphisms (DIPs) [5] or, in the case of sex-mismatched transplantation, Y-chromosome specific sequences [6], has a much better sensitivity (≤0.1%). An inherent restraint of qPCR-based techniques is their comparatively low accuracy in the state of mixed chimerism (i.e., <90% donor cells). We and others have previously shown that this limitation can be overcome by a further advancement of qPCR, namely digital PCR (dPCR), which combines the excellent sensitivity of qPCR with the high accuracy and reproducibility of STR [7,8].

The principle of dPCR is based on dividing the whole PCR mix into thousands of identical small “mini-reaction” compartments, before thermocycling takes place. Thus, the template, e.g., genomic DNA, becomes diluted with the consequence that individual compartments may or may not contain (at least) one template. All compartments are amplified in parallel but separately, and successful amplification (i.e., presence of the template in the given compartment) is detected by endpoint analysis based on fluorescence detection; individual positive and negative mini-reactions can be counted. The use of Poisson statistics enables quantification of absolute target numbers present in a sample [9].

In 1999, Vogelstein and Kinzler showed that dPCR represents a promising technique for cancer genomic content determination [10]. However, only in the last few years dPCR has been broadly introduced in molecular diagnostics in different areas of medicine [11,12,13]. In hematology, several studies have indicated applicability of dPCR for the detection of minimal residual disease using a variety of molecular markers [14,15,16,17]. As noted above, principal suitability of dPCR for analysis of hematopoietic chimerism after allogeneic SCT has also been shown [7,8]. However, in order to be useful as every-day diagnostic tool for ideally all SCT patients, a broad collection of dPCR assays needs to be available, which detect an adequate number of DIPs distributed over the whole genome.

In this work, we aimed at introducing such comprehensive dPCR panel for regular chimerism diagnostics. In order to ensure precise quantification, all individual reactions had to be suitable for duplex PCR against both a reference gene and, in case of sex-mismatched SCT, a Y-chromosome specific PCR. Twenty-nine individual DIP, the reference and Y-chromosome specific assays were optimized and technically validated, before they were tested on artificial serial dilutions. Thereafter, we performed clinical validation on retrospective patient samples in direct comparison with STR analysis. Finally, we designed a screening plate that facilitates identification of suitable markers for a given recipient–donor pair in an easy and comfortable way.

## 2. Results

### 2.1. Assay Design

The aim of this project was the establishment of a broad panel of dPCR-based assays for chimerism analysis. For convenient use in every-day diagnostics, such panel has to meet several conditions: (i) all reactions should proceed under the same conditions (identical temperatures, buffers, etc.); (ii) each individual assay should be compatible with each other to allow for duplex-PCRs; and (iii) results should be easily interpretable with clear separation of positive and negative events. However, the clearly most important requirement was the inclusion of multiple alleles at different chromosomes to ensure broad coverage of ideally any donor/recipient constellation, but identical twins.

As we have shown previously [8], both donor and recipient cells can be quantified simultaneously in the same reaction using duplex PCR. Such approach is evidently optimal for chimerism analysis, but given the above requirements very difficult in practical terms. In fact, to achieve broad coverage of possible donor/recipient constellations, individual assays for approximately 30 DIPs are required (see below). If each individual assay should be combinable with each other, the total number of potential combinations would equal 435. As one would expect (and as we have proven empirically, see list of markers not included in the final panel in Supplementary Materials Table S1), not all of these combinations do harmonize in duplex PCRs, in particular if conditions cannot be adapted to individual assays.

Therefore, we decided to use an alternative strategy. We reasoned that it is much straighter to optimize all individual DIP-directed dPCR assays for duplex PCR with two different reference genes. One “universal” reference (REF) gene, *β-Globin*, can always be applied for duplex-PCR. In contrast, the second, Y-chromosome specific reference (Sex-determining region of Y gene, *SRY*) will only be useful if at least one of the two parties, donor or recipient is male, i.e., in approximately 75% of the cases. Importantly, however, after sex-mismatched SCT, inclusion of the SRY assay allows simultaneous quantification of donor vs. patient cells in the duplex PCR.

We identified an initial set of 53 human biallelic deletion-insertion polymorphisms (DIPs/Indels) by searching the RefSeq-, UniSTS-, dbSNP- and Online Mendelian Inheritance in Man (OMIM) databases [18]. We applied the following conditions: (i) markers had to be on different chromosomes or at least in a physical distance of 10 Mbp to avoid loss of information due to their linkage; (ii) allele frequencies in the published population were 40%–60%; (iii) levels of heterozygosity averaged 30%–50%; and (iv) none of the selected DIP loci had a known association with diseases or other phenotypes according. Flanking sequence of the selected DIPs were checked for sequence variants and repeat structures. For all these loci, dPCR assays were designed (Supplementary Materials Table S1).

### 2.2. Definition of Optimal Assay Conditions

As stated above, the first task was to establish uniform assay conditions that ensure high specificity and sensitivity of duplex PCRs for all possible combinatory assays, i.e., ideally several dozens of different duplex assays (DIPs against each the universal REF and the SRY assays and SRY against the universal REF).

To this aim, we adjusted a number of parameters to ensure optimal results in duplex PCR. Those included the annealing temperature, primer and probe lengths and concentrations for each single DIP-specific dPCR, but also the use of different numbers and types of quenchers.

**Figure 1:**
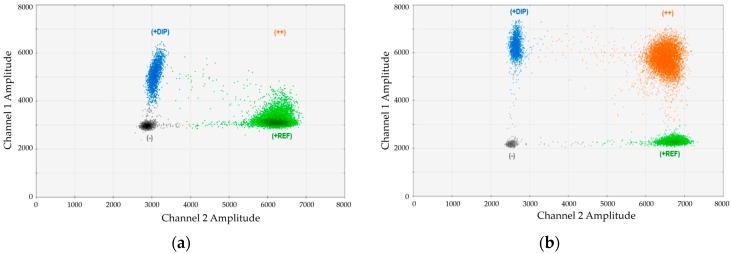
Adjustment of MgCl_2_ concentration results in substantially improved cluster separation in digital-PCR (dPCR) duplex assays: (**a**) duplex-assay DP134-I (channel 1, FAM) + REF (reference; channel 2, HEX) with the standard concentration of MgCl_2_ contained in the Bio-Rad buffer. No separation of double and single HEX-positive signals; and (**b**) the same duplex assay DP134-I + REF with optimized MgCl_2_ concentration.

Since we realized that the magnesium chloride concentration in the standard Bio-Rad (Hercules, CA, USA) buffer was not optimal for our duplex assays, we performed titration experiments adding different amounts of MgCl_2_. An example for the impact of MgCl_2_ is provided in Figure 1. In this case, we analyzed an artificial mixture of DNA from one DP134-I positive and one DP134-I negative healthy donors and performed duplex-dPCR using marker DP134-I combined with the universal reference gene (REF). Duplex PCRs were carried out using (Figure 1a) standard buffer or (Figure 1b) buffer containing an optimized magnesium-chloride concentration. As evident, the cluster of double positive droplets became only visible using the optimized conditions.

### 2.3. Technical Validation

**Figure 2:**
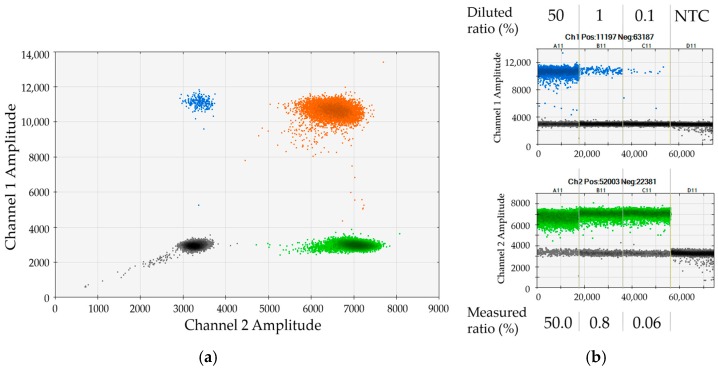
Performance of assay DP70-I in duplex dPCR with SRY (*Sex-determining region of Y* gene): (**a**) 2D dot plot displaying results of duplex dPCR combining assays DP70-I (FAM, channel 1) vs. SRY (HEX, channel 2). All four clouds representing double-negative, single-positive and double-positive droplets, respectively, are excellently separated; (**b**) 1D dot plots displaying dPCR-based quantification of serial dilutions of female DP70-I positive mononuclear cells (MNCs) in male DP70-I negative MNCs using the two markers DP70-I and SRY. As indicated, measured ratios of cells are in very good agreement with expected values.

Next, we assessed applicability of the 53 DIPs (see Section 2.1) for duplex dPCR analyses that simultaneously measure one recipient- or donor-specific marker in combination with the REF or the SRY assay. We identified 29 DIPs that showed good performance in duplex dPCRs with both REF and SRY. Some of the assays were further optimized, e.g., through primer-length modification. The genetic loci addressed by the 29 selected assays were distributed over 15 chromosomes, thus facilitating high power of discrimination. For testing of individual dPCR assays, we made use of 33 different artificial serial dilutions of mononuclear cells (MNCs) from each two healthy donors. Each single assay was analyzed in combination with the REF gene and the SRY gene using at least one of the artificial mixtures, as exemplified in Figure 2. In the given representative example, a serial dilution of female DP70-I positive cells in male DP70-I negative background was applied to validate the assay for marker DP70-I in combination with SRY. The 2D dot plot shows very good separation of the four clusters (Figure 2a). The determined chimerism was in good agreement with the expected values (Figure 2b). Please note that small discrepancies between measured and expected ratios most probably reflect minor cell-counting and pipetting variations (compare to Section 2.4).

Finally, robustness of all individual dPCR assays against variations in several parameters was verified. In particular, we assessed potential deviations in DNA input, applied reaction volumes and PCR cycle numbers, but also the possible impact of the used PCR thermocycler. We found that a minimal amount of 1 ng gDNA still ensured precise and reproducible quantification, albeit, as expected, at lower resolution. Moreover, the assays were very robust with regard to reduced reaction volumes (down to 17 µL instead of 20 µL) that might be, for example, due to not correctly calibrated pipettes. PCR cycle numbers between 35 and 45 warranted essentially identical results; we would suggest applying at least 40 cycles. Finally, we tested six thermocyclers and found excellence data coherence for our assays independent of the used device (all data are summarized in Supplementary Materials Tables S2 and S3).

### 2.4. Sensitivity and Reproducibility

We next addressed potential sensitivity of the dPCR approach. With regard to the actual sensitivity of DNA-based chimerism analysis, it is important to take into account that 1 µg of human genomic DNA corresponds to approximately 150,000 diploid genomes.

We first determined the limit of blank (LOB) for all assays (Supplementary Materials Table S4). Further on, we considered a given sample marker-positive, if at least three to five positive droplets (depending on the LOB) were detected. Consequently, the regular use of 100 ng gDNA per reaction warranted a theoretical limit of detection of at least 0.03%. If low amounts of gDNA are used (10 ng or less), e.g., early after transplantation, exact quantification will still be possible, but sensitivity will decrease accordingly (for 10 ng: 0.3%, for 1 ng: 3%).

**Figure 3:**
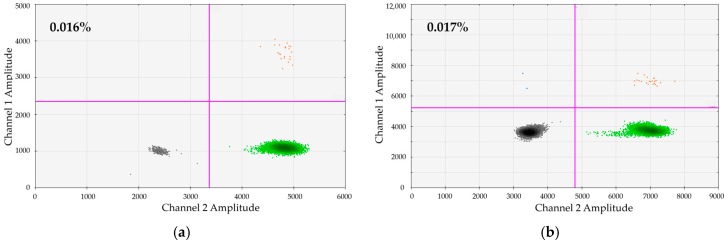
High sensitivity of dPCR-based chimerism detection. In the shown example, an artificial dilution of 0.01% MNCs positive for the marker DP114-I/DP131-I was generated in DP114-I-/DP131-I-negative MNCs. Six hundred nanograms of gDNA were subjected to duplex-dPCR using assays: (**a**) DP114-I (FAM, channel 1) vs. SRY (HEX, channel 2); and (**b**) DP131-I (FAM, channel 1) vs. Ref (HEX, channel 2).

We wanted to test whether dPCR would principally facilitate a sensitivity of 0.01% on a regular basis. To test this, we generated and tested artificial dilutions of 0.01% for 25 DIP + REF/SRY combinations. We submitted 200–600 ng gDNA to single dPCRs. In the representative examples shown in Figure 3 we analyzed a manufactured dilution of 0.01% of DP114-I/DP131-I positive in DP114-I/DP131-I negative MNCs (from healthy donors). Six hundred nanograms gDNA were subjected to dPCR. As demonstrated, very clear-cut populations of DP114-I-positive and DP131-I-positive droplets can be distinguished.

To address reproducibility and precision of dPCR assays, we carried out independent analyses for the same dilution samples using assays based on different DIPs. Data are summarized for one representative example in Table 1. The particular serial dilution shown there was analyzed with twelve different DIP-marker combinations. Please note that the means of the measured ratios show very low standard deviations indicating excellent assay performance irrespective of the used DIP marker. Moreover, the measured values as well as their means slightly differ from the expected ratios that were based on dilution factors. This indicates that the dPCR-based measurement is actually very precise, whereas variabilities are most probably introduced by cell-counting and pipetting errors.

A further indicator of reliability of an assay is its (intra-assay) reproducibility. We have previously shown excellent repeatability of dPCR in the context of chimerism determination [8]. Here we performed multiplicate dPCR assays on aliquots from identical samples in six different thermocyclers and found very high conformity for all tested assays (Supplementary Materials Table S3).

### 2.5. Technical Accuracy and Reproducibility of dPCR Assays

As evident in Table 1, dPCR-based chimerism assessment ensures high inter-method reproducibility. At the same time, as for any detection method there is some intrinsic variability. In addition, due to statistical variations, e.g., based on Poisson distribution, additional errors will be introduced at very low levels of chimerism accidentally. To address the variability in dPCR results inherent to the used assay, we evaluated more than 90 different duplex assays performed in the course of donor/recipient typing (see Section 2.7). In those assays, allele copy numbers were simultaneously measured in the same sample using two different dPCR assays that recognized different DIPs and/or REF genes. We reasoned that the deviations between the allele copy numbers determined by the two different assays would be indicative for the potential technical variations associated with our approach (i.e., duplex PCR on relatively large amounts of genomic DNA). Obtained data are summarized in Supplementary Materials Table S5. It demonstrated that the variability between the two measured values was generally very low. Indeed, the mean delta between the two measured values is 4.9% (range: 0%–26.3%), the median is 3.6%. In line with these data, for the vast majority of measurements (more than 90%), variations between the two assays were below 10%, and they reached approximately 25% in two of the assays (2.1%).

### 2.6. Clinical Evaluation and Application

To verify applicability of our panel for diagnostics, we finally assessed chimerism on patient samples post allo-SCT by both STR and dPCR. We analyzed 21 patients (9 female and 12 male), who underwent allogeneic SCT in the Dept. of Stem Cell Transplantation of the University Medical Center Hamburg-Eppendorf between 2008 and 2015. Median patient age at transplantation was 58 years (range from 39 to 74 years). Further patients’ characteristics are summarized in Table 2. The clinical study was approved by the Ethics committee of the Chamber of physicians of the city and state of Hamburg, Germany (approval code PV4942) on 19 May 2015.

**Figure 4:**
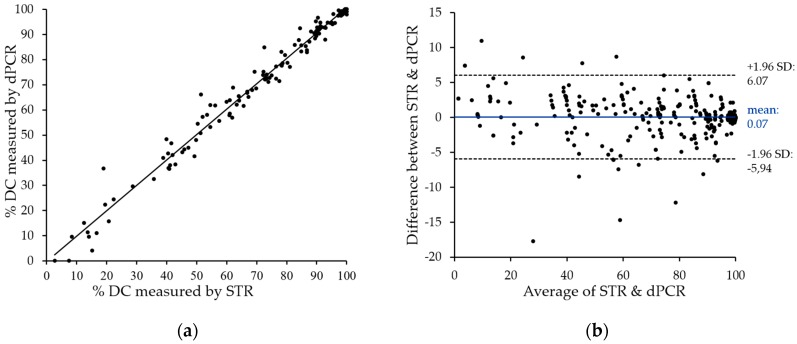
Excellent correlation of chimerism data as obtained on clinical samples by dPCR vs. STR Chimerism was assessed for 147 post-SCT samples by both dPCR and STR analyses. (**a**) Obtained data were analyzed for linear regression of percent donor chimerism (DC) (digital PCR assays are regularly designed to quantify remaining patient cells). As shown, a very high degree of correlation (*r*^2^ > 0.98) was observed; (**b**) Bland–Altmann analysis of dPCR as compared to STR data. The difference between STR and dPCR quantification is plotted against their mean. Ninety-five percent limits of agreement have been computed by average difference ±1.96× standard deviation (SD) of difference.

After screening of donor and recipient pairs for suitable DIP markers, duplex dPCR were carried out and analyzed using the QX100 PCR System (Bio-Rad) in accordance with the manufacturer’s recommendations. Results were compared with data obtained using the current “gold standard”, i.e., STR analysis, as shown in Figure 4a. The two methods showed very close correlation with an *r*^2^ value above 0.98, with only a few divergences in both directions. The only striking differences represent two samples with low, but detectable donor chimerism by STR, which are negative by patient-cell directed dPCR. These can be explained by the technical variations of dPCR in this measurement range (see Section 2.5). It is therefore mandatory to directly quantify donor cells in cases of low donor chimerism.

To visualize the degree of agreement between STR and dPCR data, we performed Bland–Altman analysis. The observed mean value of the difference (0.07%) and the agreement range (95% limit of agreement) from −5.94% to 6.07% underline the close correlation of data obtained with the two techniques.

Notably, usefulness of the dPCR assays for clinical diagnostics was also proven by successful participation in standardized External Quality Assessment Schemes performed by Instand e.V. (Düsseldorf, Germany).

### 2.7. Screening Plate and Test of Discriminative Power

The final dPCR panel that passed all quality checks comprises 30 markers (29 DIPs and the sex-typing locus). In the last step, we developed a screening plate for initial genotyping with DIP-specific duplex dPCR assays in order to facilitate convenient use in routine diagnostics. Using this screening plate, informative DIPs can be easily determined for a given donor/recipient constellation.

The loci targeted with the new dPCR panel are distributed over 15 chromosomes, which in theory should warrant a high power of discrimination. To address this question empirically, we investigated all 176 adults transplanted in our department in 2015 for the presence of the respective DIPs. We identified at least one feasible duplex-PCR assay for 174 of the 176 donor/recipient pairs, corresponding to 98.9%, proving the very good discriminative power of the panel.

## 3. Discussion

Chimerism monitoring is a crucial diagnostic measure after allogeneic SCT, and STR analysis is the “gold standard” to measure chimerism at present. For quantification, STR relies on capillary electrophoresis broadly used for Sanger sequencing, but nowadays threatened with extinction. Moreover, based on the currently available read-out technique, usefulness of STR is constrained by its low sensitivity of 1% to 5% [3,19]. This might be particularly relevant when late rejection or an impending relapse require early therapeutic intervention. In those cases, a comparatively robust, but more sensitive method would be of great advantage.

More than a decade ago, real-time quantitative PCR based on a Y-chromosome specific sequences and InDels [3,19] was introduced for chimerism assessment, which confers sensitivities of at least 0.1%. However, based on the underlying quantification approach, qPCR results show relatively high variability in cases of mixed chimerism (10%–90%). Although accuracy can be assured using replicates and quantifying both donor and recipient cells, qPCR has not yet become commonly used.

Recently, others and we have shown that a further development, digital PCR has the potential to overcome limitations of qPCR [7,8]. Indeed, dPCR-based chimerism analysis combines the excellent sensitivity of the qPCR with the high accuracy and reproducibility of the STR. However, initial proof-of-principle studies were performed with only limited sets of markers, which would not suffice to offer this diagnostic tool to (ideally) all transplanted patients [7,8].

To address this need, we here aimed at developing a whole panel of assays detecting different DIPs that had to cover multiple regions of the human genome to ensure maximal discriminative power. Obviously, this goal ideally requires a maximal set of assays. At the same time, our idea was to develop an easy-to-use kit, where all individual reactions can be performed with the same buffer and using the same amplification program. This was particularly challenging, since all assays had to be efficient in duplex dPCRs, in order to ensure accurate quantification. Indeed, of the 53 DIP-specific assays initially tested, only 29 successfully underwent all optimization and validation steps. This means that all 29 assays can be performed under the specified standard PCR conditions in conjunction with either the reference assay or the Y-chromosome specific assay that will be particularly useful in the context of sex-mismatched allo-SCT. In addition, the SRY assay was optimized to be carried out in duplex with the REF assay. Notably, for initial genotyping of donor–recipient pairs, we developed a screening plate that allows comfortable and safe identification of suitable dPCR assays in routine laboratories.

In our experience, in most cases it will be sufficient to quantify patient cells remaining after allo-SCT for precise chimerism determination. Only in cases of low donor chimerism, it is obligatory to directly measure the contribution of donor cells (see next paragraph). Based on the excellent inter-assay conformity (Table 1), we suggest that routine chimerism can be performed using just one (patient-) specific marker. However, the use of multiple assays might be considered in patients with high risk for chromosomal instability. Moreover, we suggest to simultaneously enumerate both recipient and donor cells, if possible. Using the panel introduced here, this can easily be done in essentially all cases of sex-mismatched SCT, i.e., for approximately 50% of all patients.

It could not be excluded a priori that the used setting (duplex dPCR on relatively high amounts of genomic DNA) might be associated with some technical measuring variability. In fact, despite our optimization strategy, PCRs ran in duplex might still influence each other, and a differential impact of the relatively high amounts of genomic DNA and potential sample contaminations (proteins, salts) analyzed on the two individual assays might not be completely excluded. Obviously, sample processing in the used droplet-digital PCR device will further increase the inherent statistical variation in the distribution of the two analyzed alleles. Finally, the emission spectra of the two fluorescence dyes used in the Bio-Rad system show some overlap and therefore require compensation, which obviously might affect results. To address technical accuracy, we simultaneously determined allele numbers in multiple samples using two different DIP-specific assays in a duplex dPCR. Notably, the median difference between the two measured values was below 4%. Thus, the methodological error of our approach cannot be expected to affect validity of data. This underlines usefulness of dPCR for chimerism analysis, particularly for the status of complete or almost complete donor chimerism with only very few remaining cells to be detected. It is important to note, however, that the observed inherent error becomes relevant, if donor chimerism falls under the level of 20% to 30%, and only recipient cells are being detected by dPCR assays (compare to Figure 4a: two values positive for donor cells by STR, but negative by dPCR). In that case, accurate determination of donor chimerism cannot be achieved solely based on quantifying recipient-specific alleles. In contrast, an assay detecting a donor-specific DIP needs to be performed in parallel to ensure precise quantification of remaining donor cells.

Since the panel has been intended for diagnostic use, sensitivity, specificity, accuracy and reproducibility represent key specifications. We used artificial serial dilutions to verify that each individual assay meets these requirements. Indeed, comparative analyses of defined samples using different assays confirmed excellent accuracy and reproducibility. For most assays (25), we also tested sensitivity using cell dilutions of 0.01% and detected the low-level cells in all but one cases. It needs to be noted, however, that increased sensitivity can only be accomplished with higher DNA amounts. If too much template is being used, the reference PCR will become saturated (all droplets become positive), which precludes correct quantification. Moreover, very high sensitivities might not be meaningful for routine chimerism analyses of blood samples, since the latter are usually “contaminated” by recipient cells (skin, endothelium) [20]. On the other hand, the contamination problem is unlikely to occur, if selected cell populations (e.g., CD34) are being analyzed.

In order to assess clinical utility of our panel, we analyzed 147 peripheral blood samples from 22 patients post allo-SCT in direct comparison with STR. Notably, we found a very high correlation between chimerism data obtained with the two different methods (*r*^2^ > 0.98) indicating that dPCR matches the reliability of STR-PCR. In addition, when we compared data on identical samples independently obtained with either different assays or repetitive measurements using the same assay, we observed excellent accuracy and reproducibility. Importantly, obtained chimerism kinetics were in excellent agreement with the clinical course of the patients included in this study (not shown), which is an essential precondition for using this parameter as a prognostic marker [21] and/or to guide clinical decisions.

In order to verify whether the number of assays included in our final panel were sufficient to find an informative locus for most if not all donor–recipient pairs, we tested applicability for all consecutive adult patients transplanted in our center in 2015 (*n* = 176). For 174 of the 176 donor–patient pairs (98.9%) we found at least one, in the vast majority several assays informative for chimerism testing. This is conform to the statistical calculations by Dwight et al. [22] that was performed for informative SNP loci. For our approach, a locus is informative, if discordantly homozygous in donor and recipient, or homozygous in one and heterozygous in the other. Based on these considerations, for 30 informative loci (including SRY) and an allele frequency down to 3%, at least one marker should be found for more than 95% of recipient/donor pairs [22]. However, in our panel we only included DIPs with allele frequencies between 40% and 60%. Thus, the very high coverage of almost 99% as observed for the tested 176 patients is in perfect agreement with the theoretical expectations.

With regard to practical application, dPCR has certain advantages as well as shortcomings in comparison with current technology. The price per sample is relatively high, although with the market entry of new players some reduction might be expected. In addition, the droplet-digital PCR device used in our study (QX100) necessitates relatively long hands-on time per sample, but automated sample-processing devices are available, which significantly reduce the amount of manual work. On the plus side, dPCR is easy to perform and does not require replicate analysis or generation of standard curves for quantification of chimerism samples. Based thereon, chimerism data can be obtained very quickly, usually within 24 h. Moreover, in most cases, existing qPCR protocols can readily be adapted to dPCR conditions.

In summary, we have established a very convenient tool for routine chimerism analysis based on digital PCR. We have shown that the panel of dPCR assays introduced here facilitates accurate and high-sensitivity chimerism assessment in patients after allogeneic SCT. Based on theoretical considerations, but also our empirical data, the panel might be applicable for almost 99% of allo-SCT patients. We conclude that our new tool represents a promising alternative technique for molecular chimerism diagnostics.

## 4. Materials and Methods

### 4.1. Patients

Twenty-one consecutive adult patients (nine female and twelve male) transplanted between 2008 and 2015 in the University Medical Center Hamburg-Eppendorf were included in the clinical follow-up study (Table 1). Median age at transplantation was 58 years (range from 39 to 74 years). Patients suffered from different diseases: MM (*n* = 2), MDS (*n* = 4), AML (*n* = 8), CML (*n* = 3), CLL (*n* = 1) and MF (*n* = 3). Peripheral blood samples were analyzed for chimerism by STR and dPCR in parallel for at least four time points after SCT. All patients agreed and gave their written informed consent for the acquisition of genetic data in the context of scientific studies.

The testing of human samples for clinical validation of the novel, dPCR-based diagnostic method was approved by the Ethics committee of the Chamber of physicians of the city and state of Hamburg (approval code PV4942) on 19 May 2015.

### 4.2. Genomic DNA

Genomic DNA was prepared from unseparated peripheral blood samples using the BioRobot M48 workstation (QIAGEN, Hilden, Germany). To generate artificial serial dilutions mononuclear cells (MNCs) of two healthy donors were used. DNA was isolated using the QIAamp DNA Blood Mini Kit (QIAGEN).

### 4.3. Short Tandem Repeat (STR) Amplification

STRs were PCR-amplified with CE-IVD certified Mentype Chimera PCR Amplification Kit (Biotype Diagnostic GmbH, Dresden, Germany) in accordance with the manufacturer´s recommendations. PCR products were quantified using a 3500 Genetic Analyzer (Thermo Fisher Scientific Inc., Waltham, MA, USA). The ratio was calculated using the software package Chimeris Monitor (Biotype Diagnostic GmbH). Only informative donor and recipient alleles that are separated by at least *n* ± 2 alleles outside stutter region were used for calculation.

### 4.4. Digital PCR (dPCR)

Digital PCR was carried out with the QX100 and QX200 Droplet Digital PCR System (Bio-Rad, Laboratories, Hercules, CA, USA) in accordance with the manufacturer’s recommendations. Briefly a set of 30 duplex assays as dried mixes on a 96-well plate (Mentype DigitalScreen, Biotype Diagnostic GmbH) were available for the initial marker screening. For analysis of chimerism after marker screening, 29 DIPs in combination with a reference gene (β-Globin) or (for sex-mismatched SCT) a Y-chromosome specific locus and additionally the Y-chromosome specific marker with reference gene from the Mentype DigitalQuant Kit (Biotype Diagnostic GmbH) were used. Final dPCR reaction mixtures of 20-µL volume consisted of 10 μL 2× ddPCR Supermix (Bio-Rad), 2 µL 10× DigitalQuant Primermix (after optimization of primer and probe concentrations), 0.5 μL FastDigest EcoRI (Thermo Fisher Scientific, Waltham, MA, USA) and, if not indicated otherwise, about 100 ng DNA. To exclude unwanted restriction, absence of the EcoRI recognition site in all amplicons was verified based on the DNA sequences. The PCR mix was compartmentalized using the QX100/200 Droplet Generator. The produced droplet-containing water-in-oil emulsion (40 µL) was transferred to a 96-well PCR plate (Eppendorf, Hamburg, Germany), sealed and amplified in a standard thermal cycler (Mastercycler gradient, Eppendorf): denaturation (95 °C for 10 min), amplification cycles (94 °C for 30 s, 62 °C for 1 min; 45 times), ramp rate of 1.0 K/s, and final 10 min inactivation step at 98 °C. Afterwards, individual wells were analyzed simultaneously for FAM and HEX fluorescence using the QX100/200 droplet reader following the manufacturer’s instructions. Data were processed with QuantaSoft software (Bio-Rad). The mean concentration of target sequences (copies per microliter) was calculated using Poisson Algorithm incorporated in the software.

## Figures and Tables

**Table 1:** Parallel analyses of chimerism for identical DNA samples using different dPCR assays.

Serial Dilution (%)	Ratio with Different DP-Marker (%)												Mean Ratio (%)	SD
	70-D/R	70-D/Y	88-I/Y	114-I/R	114-I/Y	128-D/R	128-D/Y	131-I/R	131-I/Y	133-I/R	133-I/Y	152-D/Y		
0.01	0.014	0.021	0.016	0.019	0.016	0.022	n.d.	0.017	0.017	0.018	0.013	0.021	0.018	0.0029
0.1	0.14	0.16	0.12	0.1	0.12	0.12	0.16	0.15	0.14	0.12	0.19	0.13	0.14	0.025
1	1.2	1.2	1.2	1.2	1.1	1.3	1.1	1	1.1	1.2	1.2	1.2	1.2	0.078
50	50.7	52.7	51.5	50.6	45.5	51.3	52.2	46	49.7	47.2	52.9	50	50.0	2.51

SD, standard deviation.

**Table 2:** Characteristics of patients included in the study.

No	Sex/Age	Disease	Donor Type	STR Marker	dPCR Marker
1	f/61	AML	MUD/PBSCT	D12S391, D4S2366, D18S51, D21S2055, SE33	DP101-I/R, DP101-I/Y
2	m/49	AML	MRD/PBSCT	D18S51, D21S2055, SE33	DP140-I/R
3	m/69	MDS	MUD/PBSCT	D7S1517, D5S2500, D21S2055, SE33	DP67-D/R
4	m/63	MF	MUD/PBSCT	D12S391, D8S1132, D18S51, D21S2055, D10S2325	DP70-D/R, DP105-I/R, DP163-I/R
5	f/72	MDS	MUD/BMT	D12S391, D2S1360, D5S2500, D21S2055, SE33	DP88-I/R, DP88-I/Y, DP97-I/Y, DP131-I/Y, DP140-I/Y
6	f/58	AML	MRD/PBSCT	D18S51, SE33	DP105-D/R, DP310-I/R
7	m/65	MF	MUD/PBSCT	D7S1517, D8S1132, D21S2055	DP301-D/R
8	m/63	AML	MUD/PBSCT	AM, D3S1744, D12S391, D21S2055	DP128-D/R, DP152-D/R
9	m/44	AML	MRD/PBSCT	D7S1517, D8S1132, D21S2055, D10S2325, SE33	DP114-D/R, DP301-I/R
10	f/50	AML	MMUD/PBSCT	D7S1517, D2S1360, D18S51, SE33	DP101-D/R
11	f/66	CML	MUD/PBSCT	D7S1517, D8S1132, D18S51, D21S2055	DP131-I/R, 134-I/R, 307-I/R
12	f/57	MDS	MUD/BMT	D8S1132, D5S2500, D10S2325, SE33	DP114-I/R, DP114-I/Y, DP133-I/R, DP133-I/Y
13	m/74	MF	MRD/PBSCT	AM, SE33	DP104-I/R
14	m/55	MM	MRD/PBSCT	D12S391, D10S2325, SE33	140-I/R
15	m/58	CML	MRD/PBSCT	D2S1360, D8S1132, D21S2055, D10S2325	DP104-D/R
16	f/58	CLL	MUD/PBSCT	D12S391, D2S1360, D5S2500, D21S2055, D10S2325	DP97-I/R
17	m/57	MDS	MUD/PBSCT	D2S1360, D8S1132, D18S51, D21S2055, D10S2325	DP304-D/R, 104-D/R, 152-D/R
18	f/49	AML	MUD/PBSCT	D12S391, D21S2055, D10S2325, SE33	DP67-D/Y, DP128-D/Y, DP152-D/Y
19	f/49	CML	MUD/PBSCT	D12S391, D2S1360, D5S2500, D21S2055	DP70-I/R, DP70-I/Y
20	m/39	MM	MRD/PBSCT	AM, D3S1744, D2S1360	DPSRY/R, DP88-D/R
21	m/52	AML	MRD/PBSCT	D8S1132, D5S2500	70-I/Y, 131-I/Y

AML/CML, acute/chronic myeloid leukemia; CLL, chronic lymphatic leukemia; MM, multiple myeloma; MDS, myelodysplastic syndrome; MF, Myelofibrosis; MMUD, mismatched unrelated donor; MRD, matched related donor; MUD, matched unrelated donor; BMT, bone marrow transplantation; PBSCT, peripheral blood stem cell transplantation.

## Supplementary Materials

Supplementary materials can be found at www.mdpi.com/1422-0067/17/9/1515/s1.
