# Supplementary material for: Digital PCR Panel for Sensitive Hematopoietic Chimerism Quantification after Allogeneic Stem Cell Transplantation
Source: Int J Mol Sci. 2016 Sep 9;17(9):1515. doi: 10.3390/ijms17091515 (PMC5037792; doi:10.3390/ijms17091515)
Supplement: Supplementary file 1 [file ijms-17-01515-s001.pdf]

Supplementary Materials: Digital PCR Panel for Sensitive Hematopoietic Chimerism Quantification after Allogeneic Stem Cell Transplantation

Tanja Stahl, Caroline Rothe, Manja U. Böhme, Aloisa Kohl, Nicolaus Kröger and Boris Fehse

Table S1. Population data for the tested genetic markers (DIPs). Allele frequencies are indicated for Deletion–Insertion Polymorphisms (DIPs) tested in this study.

**DIP Locus**	**H (obs)**	**H (exp)**	**Allele Frequency** [1]									
			**German Population**		**dbSNP European**		**dbSNP Amerind**		**dbSNP Japanese**		**dbSNP African**	
			**Del.**	**Ins.**	**Del.**	**Ins.**	**Del.**	**Ins.**	**Del.**	**Ins.**	**Del.**	**Ins.**
**DP67**	0.44	0.44	**0.33** [b]	(0.67) *	**0.39**	0.61	**0.70**	0.30	**0.49**	0.51	**0.31**	0.69
**DP70**	038	0.50	**0.47**	**0.53**	**0.52**	**0,48**	**0,24**	**0.76**	0.30	0.70	0.15	0.85
**DP88**	0.54	0.50	**0.54**	**0.46**	**0.50**	**0.50**	**0.43**	**0.57**			**0.30**	**0.70**
**DP97**	0.51	0.50	0.46 [a]	**0.54**	0.46	**0.54**						
**DP101**	0.47	0.50	**0.49**	**0.51**	**0.53**	**0.47**	**0.52**	**0.48**			**0.16**	**0.84**
**DP104**	0.47	0.49	**0.59**	**0.41**	**0.58**	**0.42**	**0.51**	**0.49**			**0.56**	**0.44**
**DP105**	0.51	0.50	**0.52**	**0.48**	**0.40**	**0.60**	**0.35**	**0.65**	**0.23**	**0.77**	**0.36**	**0.64**
**DP106**	0.44	0.39	(0.27) *	**0.73**	0.26	**0.74**	0.17	**0.83**	0.16	**0.84**	0.48	**0.52**
**DP114**	0.36	0.48	**0.59**	**0.41**	**0.57**	**0.43**	**0.48**	**0.52**	**0.60**	**0.40**	**0.15**	**0.85**
**DP128**	0.45	0.50	**0.49**	(0.51) *	**0.56**	0.44	**0.74**	0.26	**0.65**	0.35	**0.35**	0.65
**DP131**	0.39	0.49	(0.44) *	**0.56**	0.44	**0.56**	0.67	**0.33**	0.54	**0.46**	0.33	**0.67**
**DP133**	0.42	0.49	0.44 [a]	**0.56**	0.53	**0.47**	0.54	**0.46**	0.24	**0.76**	0.12	**0.88**
**DP134**	0.42	0.43	(0.31) *	**0.69**	0.49	**0.51**	0.54	**0.46**	0.24	**0.76**	0.12	**0.88**
**DP140**	0.50	0.48	0.39 [a]	**0.61**	0.41	**0.59**	0.20	**0.80**	0.18	**0.82**	0.43	**0.57**
**DP152**	0.53	0.42	**0.30**	0.70 [a]	**0.35**	0.65	**0.70**	0.30	**0.66**	0.34	**0.26**	0.74
**DP163**	0.57	0.49	**0.44**	**0.56**	**0.51**	**0.49**						
**DP301**	0.53	0.50	**0.46**	**0.54**	**0.39**	**0.61**	**0.36**	**0.64**			**0.42**	**0.58**
**DP304**	0.43	0.50	**0.46**	**0.54**								
**DP307**	0.17	0.50	(0.48) *	**0.52**	0.41	**0.59**	0.52	**0.48**			0.41	**0.59**
**DP310**	0.47	0.48	(0.60) *	**0.40**	0.45	**0.55**	0.50	**0.50**			0.42	**0.58**
DP23	0.48	0.47	0.38 [a]	(0.62) *	0.37	0.63						
DP38	0.45	0.43	0.32 [a]	(0.07) *	0.46	0.54						
DP48	0.50	0.48	0.41 [a]	(0.59) *	0.50	0.50						
DP53	0.42	0.47	(0.63) *	(0.37) *	0.65	0.35	0.71	0.29	0.72	0.28	0.67	0.33
DP79	0.37	0.41	0.29 [a]	(0.71) *	0.43	0.57	0.31	0.69	0.24	0.76	0.40	0.60
DP84	0.43	0.47	(0.39)*	(0.62) *	0.45	0.55	0.50	0.50			0.22	0.78
DP91	0.63	0.49	(0.59)*	(0.42) *	0.61	0.39	0.65	0.35			0.50	0.50
DP103	0.44	0.49	(0.43)*	(0.57)*	0.49	0.51	0.26	0.74			0.28	0.72
DP110	0.43	0.49	0.42 [a]	(0.59) *	0.48	0.52	0.43	0.57	0.36	0.64	0.93	0.07
DP112	0.55	0.50	0.54 [a]	(0.47) *	0.52	0.48						
DP116	0.60	0.49	(0.56) *	(0.44) *	0.26	0.74	0.24	0.76	0.29	0.71	0.23	0.77

H (obs), observed heterozygosity; H (exp), expected heterozygosity; Del., Deletion; Ins., Insertion. [1] Allele frequencies were empirically tested for the German population. All other data are from public repositories; [a] markers were not tested; [b] DIPs included in the final panel are printed in **bold**; * removed from final marker panel.

Table S2. Technical validation of dPCR assays. Robustness of the dPCR assays was tested changing the indicated variables.

Variable	Description/Reasoning	Outcome
DNA input	Different amounts of gDNA (between 20 pg and 280 ng) in order to determine the optimal range with regard to assay performance and reproducibility.	Results were well reproducible in the range between 1 and 280 ng gDNA.
Reaction volumes	dPCR reaction volumes of 17 µL, 18 µL, 19 µL were tested in comparison to 20 µL (as recommended by the manufacturer) to address robustness against loss of reaction volumes, due to handling or not correctly calibrated pipettes.	Deviations in reaction volumes up to −3 µL did not influence dPCR results.
PCR cycle numbers	Test of the amplification protocol with 30, 35, 40, 45 cycles using different DNA amounts using a serial dilution of DNA (20 pg, 1 ng, 50 ng, 120 ng, 160 ng) to verify the impact of cycle numbers on assay results.	• 30 cycles: not in all cases sufficient to ensure separation of positive and negative droplets.<br>• 45 cycles: no further improvement of sensitivity.<br>∑: Optimal results with 35–45 cycles<br>➔ Standard: 40 cycles<br>➔ Deviation by ± 5 cycles is not critical.
Various PCR thermocycler	Test of standard protocol with following different thermocyclers:<br>(1) Bio-Rad DNA Engine PTC-200 (aluminum block), Bio-Rad Laboratories.<br>(2) Biometra T1 (silver block), Biometra GmbH.<br>(3) Eppendorf Mastercycler ep-S (silver block), Eppendorf AG, Hamburg.<br>(4) Mastercycler nexus (aluminum block), Eppendorf AG, Hamburg.<br>(5) Applied Biosystems GeneAmp® PCR System 9700 (aluminum block), Life Technology GmbH, Darmstadt.<br>(6) Applied Biosystems GeneAmp® PCR System 9700 (silver block), Life Technology GmbH, Darmstadt.<br>Aim: To assess assay compatibility with different cycler platforms.	• All evaluation criterions were fulfilled for each tested cycler (see Table S3).<br>• Further experiments indicated the necessity to adapt ramping rates in dependence on the material of the thermoblock:<br>➔ Silver block: 1 K/s<br>➔ Aluminum block: 2 K/s<br>• Excellent intra-assay conformity even with different cyclers.

Table S3. Technical validation and assay performance on various thermocyclers. Assay performance was tested independently in six different thermocyclers (for details on cyclers, see Supplementary Materials Table S2). To this end, chimerism was assessed on aliquots from defined DNA samples using replicate analyses with the same dPCR assay. Please note the high assay conformity independent of the used thermocycler.

dPCR-Assay	TC 1			TC 2			TC 3			TC 4			TC 5			TC 6		
	Criterion		Ratio	Criterion		Ratio	Criterion		Ratio	Criterion		Ratio	Criterion		Ratio	Criterion		Ratio
	sep	spec	(%)	sep	spec	(%)	sep	spec	(%)	sep	spec	(%)	sep	spec	(%)	sep	spec	(%)
DP67-D+REF	√	√	0.56	√	√	0.57	√	√	0.58	√	√	0.59	√	√	0.59	√	√	0.59
DP70-D+REF	√	√	0.99	√	√	1.00	√	√	1.07	√	√	0.97	√	√	0.91	√	√	1.02
DP70-I+REF	√	√	1.00	√	√	1.01	√	√	1.04	√	√	1.04	√	√	1.02	√	√	1.02
DP97-I+REF	√	√	0.43	√	√	0.42	√	√	0.42	√	√	0.43	√	√	0.41	√	√	0.44
DP101-D+REF	√	√	0.44	√	√	0.45	√	√	0.45	√	√	0.46	√	√	0.47	√	√	0.47
DP101-I+REF	√	√	0.39	√	√	0.37	√	√	0.38	√	√	0.37	√	√	0.37	√	√	0.37
DP105-D+REF	√	√	0.40	√	√	0.44	√	√	0.45	√	√	0.44	√	√	0.43	√	√	0.45
DP105-I+REF	√	√	0.40	√	√	0.44	√	√	0.45	√	√	0.44	√	√	0.43	√	√	0.45
DP106-I+REF	√	√	0.39	√	√	0.42	√	√	0.43	√	√	0.42	√	√	0.43	√	√	0.42
DP114-D+REF	√	√	0.35	√	√	0.33	√	√	0.32	√	√	0.31	√	√	0.33	√	√	0.31
DP114-I+SRY	√	√	0.77	√	√	0.70	√	√	0.69	√	√	0.77	√	√	0.78	√	√	0.68
DP128-D+SRY	√	√	1.30	√	√	1.31	√	√	1.36	√	√	1.42	√	√	1.29	√	√	1.40
DP131-I+SRY	√	√	2.92	√	√	2.93	√	√	2.89	√	√	2.91	√	√	2.85	√	√	2.98

TC, thermocycler; sep, clear separation of positive and negative droplets; spec, specificity; √, fulfilled.

Table S4. Limit of blank for the dPCR assays. The limit of blank (LOB) was determined using standard assay conditions and 120 ng gDNA per assay. Number of positive droplets are shown. In accordance with the LOB, the limit of detection was defined as three to five signals per assay corresponding to 1/6000 (0.017%) to 1/3600 (0.028%) for the tested amount 120 ng gDNA.

**DIP**	**dPCR-Assay**	
	**with REF**	**with SRY**
DP67-D	3	3
DP70-D	0	1
DP70-I	0	0
DP88-D	0	0
DP88-I	0	0
DP97-I	0	0
DP101-D	0	0
DP101-I	0	0
DP104-D	0	0
DP104-I	0	3
DP105-D	0	0
DP105-I	0	0
DP106-I	1	1
DP114-D	0	0
DP114-I	0	0
DP128-D	0	0
DP131-I	0	0
DP133-I	1	1
DP134-I	1	1
DP140-I	0	1
DP152-D	3	3
DP163-D	0	0
DP163-I	1	0
DP301-D	2	0
DP301-I	0	0
DP304-D	0	0
DP304-I	0	0
DP307-I	0	0
DP310-I	0	0
DPSRY	3	

Table S5. Technical Error. Two different alleles (the marker and the reference gene) were simultaneously quantified in each sample using suitable duplex assays. For homozygous markers, measured copy numbers could directly be compared with assessed numbers of reference alleles, whereas numbers measured for heterozygous marker had to be multiplied by a factor of 2 (see Column "Correction").

Concentration (Copies/µL)					
**Heterozygous Marker**	**Homozygous Marker**	**Correction**	**Reference**	**Ratio A/R**	**Delta (%)**
460		920	901	1.021	2.1
531		1062	974	1.090	9.0
474		948	918	1.033	3.3
432		864	945	0.914	8.6
450		900	906	0.993	0.7
436		872	918	0.950	5.0
443		886	938	0.945	5.5
425		850	873	0.974	2.6
470		940	904	1.040	4.0
426		852	846	1.007	0.7
452		904	931	0.971	2.9
446		892	932	0.957	4.3
437		874	918	0.952	4.8
	908	908	918	0.989	1.1
	842	842	846	0.995	0.5
	945	945	930	1.016	1.6
	906	906	924	0.981	1.9
	914	914	948	0.964	3.6
452		904	918	0.985	1.5
473		946	931	1.016	1.6
427		854	926	0.922	7.8
435		870	926	0.940	6.0
	860	860	910	0.945	5.5
463		926	923	1.003	0.3
469		938	926	1.013	1.3
437		874	912	0.958	4.2
372		744	735	1.012	1.2
	801	801	808	0.991	0.9
362		724	790	0.916	8.4
389		778	787	0.989	1.1
369		738	811	0.910	9.0
261		522	511	1.022	2.2
375		750	996	0.753	24.7
372		744	781	0.953	4.7
396		792	791	1.001	0.1
390		780	803	0.971	2.9
374		748	801	0.934	6.6
362		724	765	0.946	5.4
	752	752	817	0.920	8.0
372		744	781	0.953	4.7
404		808	830	0.973	2.7
	812	812	818	0.993	0.7
	792	792	809	0.979	2.1
391		782	796	0.982	1.8
401		802	821	0.977	2.3
397		794	792	1.003	0.3
359		718	788	0.911	8.9
356		712	782	0.910	9.0
	719	719	785	0.916	8.4
399		798	821	0.972	2.8
409		818	820	0.998	0.2
384		768	810	0.948	5.2
	1556	1556	1488	1.046	4.6
	1103	1103	1497	0.737	26.3

Table S5. *Cont.*

Concentration (Copies/μL)					
**Heterozygous Marker**	**Homozygous Marker**	**Correction**	**Reference**	**Ratio A/R**	**Delta (%)**
677		1354	1521	0.890	11.0
690		1380	1510	0.914	8.6
	1415	1415	1532	0.924	7.6
	1444	1444	1538	0.939	6.1
	1312	1312	1534	0.855	14.5
757		1514	1537	0.985	1.5
769		1538	1515	1.015	1.5
667		1334	1450	0.920	8.0
676		1352	1542	0.877	12.3
729		1458	1533	0.951	4.9
789		1578	1543	1.023	2.3
	1486	1486	1531	0.971	2.9
	1427	1427	1512	0.944	5.6
	1304	1304	1505	0.866	13.4
614		1228	1493	0.823	17.7
602		1204	1473	0.817	18.3
774		1548	1526	1.014	1.4
	435	435	436	0.998	0.2
196	392	392	439	0.893	10.7
212		424	442	0.959	4.1
208		416	430	0.967	3.3
201		402	420	0.957	4.3
210		420	420	1.000	0.0
211		422	438	0.963	3.7
213		426	429	0.993	0.7
211		422	434	0.972	2.8
	413	413	431	0.958	4.2
	413	413	433	0.954	4.6
207		414	421	0.983	1.7
206		412	420	0.981	1.9
215		430	428	1.005	0.5
	433	433	431	1.005	0.5
	419	419	431	0.972	2.8
	392	392	430	0.912	8.8
197		394	422	0.934	6.6
190		380	411	0.925	7.5
217		434	435	0.998	0.2
215		430	419	1.026	2.6
				**Mean**	**4.9**
				**Median**	**3.6**
